# Editorial: Novel Approaches to Assess Disease Dynamics at the Wildlife Livestock Interface

**DOI:** 10.3389/fvets.2019.00409

**Published:** 2019-11-22

**Authors:** Ferran Jori, Beatriz Martínez-López, Joaquin Vicente

**Affiliations:** ^1^UMR “Animal, Health, Territories, Risks and Ecosystems” (ASTRE, CIRAD-INRA-Université de Montpellier), Montpellier, France; ^2^Center for Animal Disease Modeling and Surveillance (CADMS), Department of Medicine and Epidemiology, School of Veterinary Medicine, University of California, Davis, Davis, CA, United States; ^3^Institute for Game and Wildlife Research-IREC (UCLM-CSIC), Ciudad Real, Spain

**Keywords:** wildlife, infection, transmission, dynamics, local knowledge, human practices

## Introduction

Our planet is transforming quickly and these changes generate a huge biodiversity loss and threaten nature's contribution to human livelihoods. The global human demographic growth generates a huge demand in food resources, which induce the transformation of natural habitats into agricultural land and the competition of wildlife and livestock for natural resources. These drivers of change are on the rise and we can anticipate an exponential growth of interactions between wildlife livestock and people, with important implications in terms of environmental conservation, biosecurity, livestock production and disease emergence (1). From the disease perspective, the risk of unexpected events resulting from wildlife populations trying to adapt to a changing world have never been so real. This is confirmed by the global spread of animal “pandemics” such as Avian Influenza or African swine fever, which generate high mortality in wildlife and/or livestock, reduce productivity and trade, increase food insecurity and in some case seriously impact on public health (2). Therefore, the need for understanding the drivers and dynamics of this interface between domestic, wild animals is bigger than ever. This is a great challenge because this interface represents critical points for cross-species disease transmission and emergence of pathogens that are complex, difficult to monitor and require innovative, interdisciplinary, approaches and coordinated actions to improve disease prevention and management.

This Research Topic features a series of examples illustrating the complexity of wildlife-livestock interface ecosystems and the diversity of tools and methodologies applied in the study of disease dynamics in a variety of geographical areas, including Asian, African, and European settings.

## Diversity of Wild-Domestic Interfaces

Our Research Topic illustrates this diversity by the presentation of three papers focusing specifically on interactions between wild and domestic suiform species using different monitoring methods in Africa (Payne et al.) and Europe (Jori et al.; Charrier et al.). This interface is poorly covered in the scientific literature until recently, and is particularly topical and timely, considering the spread of African swine fever in those two continents. However, it is also relevant for many other shared pathogens circulating in the domestic and wild pig population such as Aujeszky's disease and Hepatitis E (Charrier et al.), respectively. Two other papers focus on the influence of transformed ecosystems in the dynamics of Avian Influenza viruses in Asian (Gilbert et al.) and Mediterranean habitats (Bárbara et al.). Another two studies present different applications of the use of camera traps to monitor direct interactions between multi-taxon mammal hosts and vectors (Hofmeester et al.) or indirect interactions through the management of big game remains, in hunting estates from South Central Spain (Carrasco-Garcia et al.). Despite a large geographic diversity, most of the areas covered represent ecosystems with an important anthropic influence.

## A Panel of Tools for Understanding New Interface Hotspots

The study of interactions among sympatric host species has aroused interest in epidemiology as an approach implemented to detect effective contacts involving pathogen transmission within communities of hosts. The study of epidemiological interactions has been a relevant topic in applied sciences for many years. However, interactions with a potential risk of pathogen transmission are multiple and complex, and many of the approaches employed to monitor these processes have been developed and adapted from other fields, mainly in the last two decades. Understanding this complexity requires the implementation of different tools and methods to assess aspects linked with the environment, its pathogens and the species that share the studied interface, including humans. Some of the papers presented in this collection, characterize the interface by describing or measuring ecological interactions between sympatric species. The use of camera traps, to monitor interactions between sympatric species is not new. However, in this Research Topic several papers use this method in different innovative contexts such as wildlife visits in rural crops from Uganda (Payne et al.), the scavenging behavior of wildlife species on hunting offal in Spain (Carrasco-Garcia et al.) or for simulating interactions between ticks and their wild and domestic ungulate hosts in the Netherlands (Hofmeester et al.). In some studies, different ecological approaches are combined with pathogen or parasite surveys and statistical or spatial modeling, providing innovative approaches to characterize the host community (Hofmeester et al.). or the influence of land use changes in the emergence of avian influenza (Gilbert et al.).

## Understanding the Human Factor

Our collection addresses on several occasions the important influence of human behavior in the development of new interfaces and the occurrence and distribution of infectious diseases in this context. The transformation of what were once natural habitats into productive areas, facilitates contacts between phylogenetically close species, such as wild and domestic pig species in Uganda (Payne et al.) and Corsica (Charrier et al.; Jori et al.). In other instances, human population growth generates increasingly vast areas of landfills (Bárbara et al.) or the expansion of rice paddy fields (Gilbert et al.), which attract various species of aquatic bird hosts, facilitating infectious contacts for the transmission of avian influenza. In a similar way, intensive hunting management creates opportunities for disease maintenance, highlighting the importance of removing hunting offal from the environment for preventing potential dissemination of diseases between sympatric wild ungulates (Carrasco-Garcia et al.; Jori et al.; Charrier et al.). The impact of human practices on the wildlife-domestic interface is assessed through different approaches such as modeling the temporal and spatial spread of rice paddy fields in Asia (Gilbert et al.), estimating the burden of disease in wild boar populations exposed to pig farming (Charrier et al.) or the production of urban waste (Bárbara et al.). In other contexts, knowledge of local communities as privileged observers of wildlife-livestock interactions, is collected through semi-structured questionnaires (Jori et al.; Payne et al.).

## Final Remarks

The concept of interface in all its dimensions is complex and susceptible to change together with the evolution of natural landscapes, but specially, with increasing human intervention (or impact). One of the characteristics of the wildlife-livestock interface is the integration of ecological, agricultural and human systems, which requires the need to consider multiple and diverse disciplines and solutions. This Research Topic illustrates the challenge to assess and control health problems at the wildlife-livestock-human interface and the need to adopt innovative approaches that merge academic disciplines with social aspects and engage relevant partners.

Many of the articles around the wildlife-livestock interface topic addressed the spread of diseases affecting livestock (avian influenza, pig diseases, or tick borne diseases). As identified in previous review on the topic (1), there is a bias toward anthropocentric funded driven priorities already identified in the literature Future article collections are needed to address this gap and explore the other side of the coin, represented by disease transmission in wildlife populations and its potential impact in biodiversity.

## Author Contributions

FJ has written the manuscript. BM-L and JV have read the manuscript, made corrections and approved it for publication.

### Conflict of Interest

The authors declare that the research was conducted in the absence of any commercial or financial relationships that could be construed as a potential conflict of interest.
